# Automated diagnosis of optical coherence tomography imaging on plaque vulnerability and its relation to clinical outcomes in coronary artery disease

**DOI:** 10.1038/s41598-022-18473-5

**Published:** 2022-08-18

**Authors:** Hirohiko Niioka, Teruyoshi Kume, Takashi Kubo, Tsunenari Soeda, Makoto Watanabe, Ryotaro Yamada, Yasushi Sakata, Yoshihiro Miyamoto, Bowen Wang, Hajime Nagahara, Jun Miyake, Takashi Akasaka, Yoshihiko Saito, Shiro Uemura

**Affiliations:** 1 Institute for Datability Science, Osaka University, Suita, Japan; 2 Department of Cardiology, Kawasaki Medical School, 577 Matsushima, Kurashiki, Okayama 701-0192 Japan; 3 Cardiovascular Medicine, Wakayama Medical University, Wakayama, Japan; 4 Cardiovascular Medicine, Nara Medical University, Kashihara, Japan; 5 Cardiovascular Medicine, Osaka University Graduate School of Medicine, Suita, Japan; 6 Open Innovation Center, National Cerebral and Cardiovascular Center, Suita, Japan; 7 Graduate School of Engineering, Osaka University, Suita, Japan

**Keywords:** Medical research, Cardiovascular biology, Data processing

## Abstract

This study sought to develop a deep learning-based diagnostic algorithm for plaque vulnerability by analyzing intravascular optical coherence tomography (OCT) images and to investigate the relation between AI-plaque vulnerability and clinical outcomes in patients with coronary artery disease (CAD). A total of 1791 study patients who underwent OCT examinations were recruited from a multicenter clinical database, and the OCT images were first labeled as either normal, a stable plaque, or a vulnerable plaque by expert cardiologists. A DenseNet-121-based deep learning algorithm for plaque characterization was developed by training with 44,947 prelabeled OCT images, and demonstrated excellent differentiation among normal, stable plaques, and vulnerable plaques. Patients who were diagnosed with vulnerable plaques by the algorithm had a significantly higher rate of both events from the OCT-observed segments and clinical events than the patients with normal and stable plaque (log-rank p < 0.001). On the multivariate logistic regression analyses, the OCT diagnosis of a vulnerable plaque by the algorithm was independently associated with both types of events (p = 0.047 and p < 0.001, respectively). The AI analysis of intracoronary OCT imaging can assist cardiologists in diagnosing plaque vulnerability and identifying CAD patients with a high probability of occurrence of future clinical events.

## Introduction

Despite the advances in medical science and healthcare practice, patients presenting with acute coronary syndrome (ACS) or with a history of myocardial infarction (MI) still have a significantly high rate of recurrent cardiovascular events. In particular, more than 20% of ACS patients who were successfully treated by percutaneous coronary intervention (PCI) will have secondary cardiovascular events within 3 years due to the worsening of previously treated culprit lesions or the progression of untreated nonculprit coronary plaques^1^. More recently, the PROSPECT II study showed that 13.2% of recent (within the past 4 weeks) MI patients who were treated by successful PCI had adverse events within 4 years^2^.

A vulnerable plaque is defined as a precursor coronary atherosclerotic plaque that may cause future coronary events by plaque rupture and subsequent intraluminal thrombosis^3^. A vulnerable plaque is generally characterized as a plaque with typical pathohistological features such as a large amount of lipid accumulation, inflammatory cell infiltration, intraplaque angiogenesis, and the presence of a thin fibrous cap that covers a lipid core. Coronary angiography is, however, not able to characterize the plaque component and the vulnerability of the plaque. The use of intravascular imaging is one recent approach to this problem. Optical coherence tomography (OCT) is a novel intravascular imaging modality with a high spatial resolution that is comparable with the histopathology, and vulnerable plaques, which are diagnosed by OCT, have been shown to be associated with the subsequent progression of coronary artery stenosis, as well as future major adverse cardiac events^4,5^. For the secondary prevention of coronary artery disease (CAD), patients may have the opportunity to have their coronary lesions evaluated directly by intravascular imaging. However, the detailed analysis of OCT images in daily practice is difficult to perform because of the large numbers of OCT images that need to be evaluated. In addition, OCT image interpretation requires a highly skilled cardiologist due to the complex morphological configurations of the lesions and the coexisting imaging artifacts. To realistically address these problems, a possible solution is the application of artificial intelligence (AI) for the image analysis.

The purposes of the study were (1) to develop a deep learning-based diagnostic algorithm for coronary plaque vulnerability by analyzing intravascular OCT images; (2) to test the diagnostic accuracy of the algorithm for plaque vulnerability; and (3) to investigate the relation between AI diagnosis of plaque vulnerability and clinical outcomes by using OCT-observed segments from patients with CAD.

## Methods

### Study population

The study patients were recruited from the OCT clinical database that was obtained from three university hospitals in Japan: Kawasaki Medical School, Nara Medical University, and Wakayama Medical University. From these databases, patients who underwent intracoronary OCT imaging during coronary angiography or PCI from 2010 to 2019 were screened (n = 6625) and were enrolled in this study when they fulfilled the following criteria: (1) patients who underwent OCT imaging of nonculprit lesions if they were diagnosed to have clinically overt CAD and (2) patients who had OCT imaging of angiographically normal or minor stenosis (< 25%) segments if they had no significant coronary stenosis. Patients who did not have OCT imaging of nonculprit lesions (n = 3735) were excluded from this study. Patients with poor OCT image quality or with severe calcification (n = 1099) were also excluded from this study.

**Figure 1:**
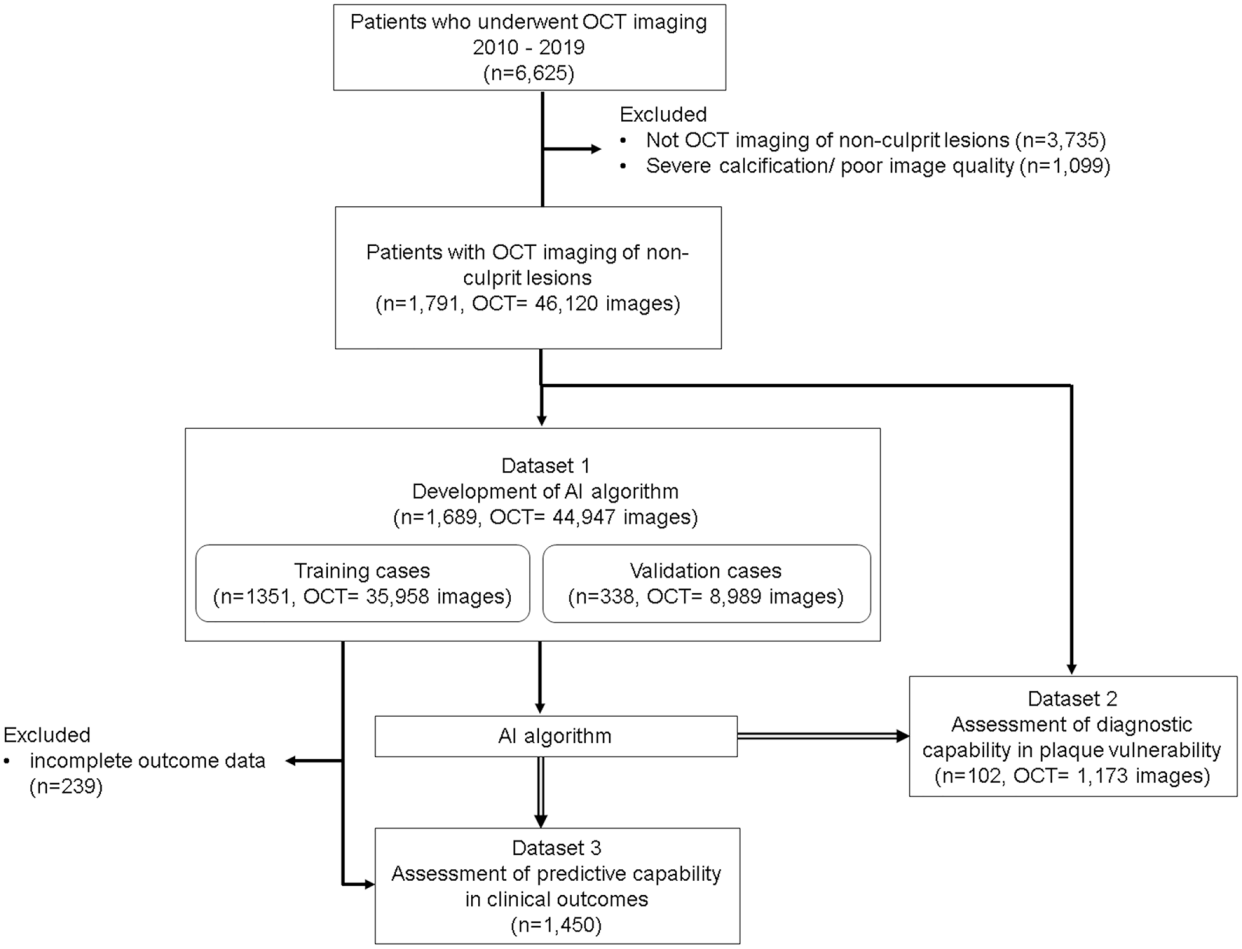
Study flow chart. *OCT* optical coherence tomography, *AI* artificial intelligence.

From the database, 1791 patients whose OCT examinations matched the inclusion criteria were eventually identified, and they were randomly assigned to dataset 1 for the development of the AI algorithm (n = 1689, training: validation = 8:2) and to dataset 2 for the testing of the developed program (n = 102). In dataset 1, 1450 patients who had complete long-term clinical outcome data for at least one month after the index OCT examination were enrolled in the follow-up study (dataset 3). The study flow chart is shown in Fig. 1.

This study complied with the Declaration of Helsinki and was approved by the institutional ethical review board of Kawasaki Medical School (IRB number: 3438-1). Written, informed consent was waived by the institutional review board (ethical review board of Kawasaki Medical School) because of the retrospective design of the study; however, informed consent was obtained in the form of an opt-out on the website. Those patients who rejected consent to the study were excluded. This study was called the TACUMI study (The Automated diagnosis of Coronary vUlnerable plaque using Medical artificial Intelligence).

### Selection of the coronary segment for analysis and OCT image analysis

**Figure 2:**
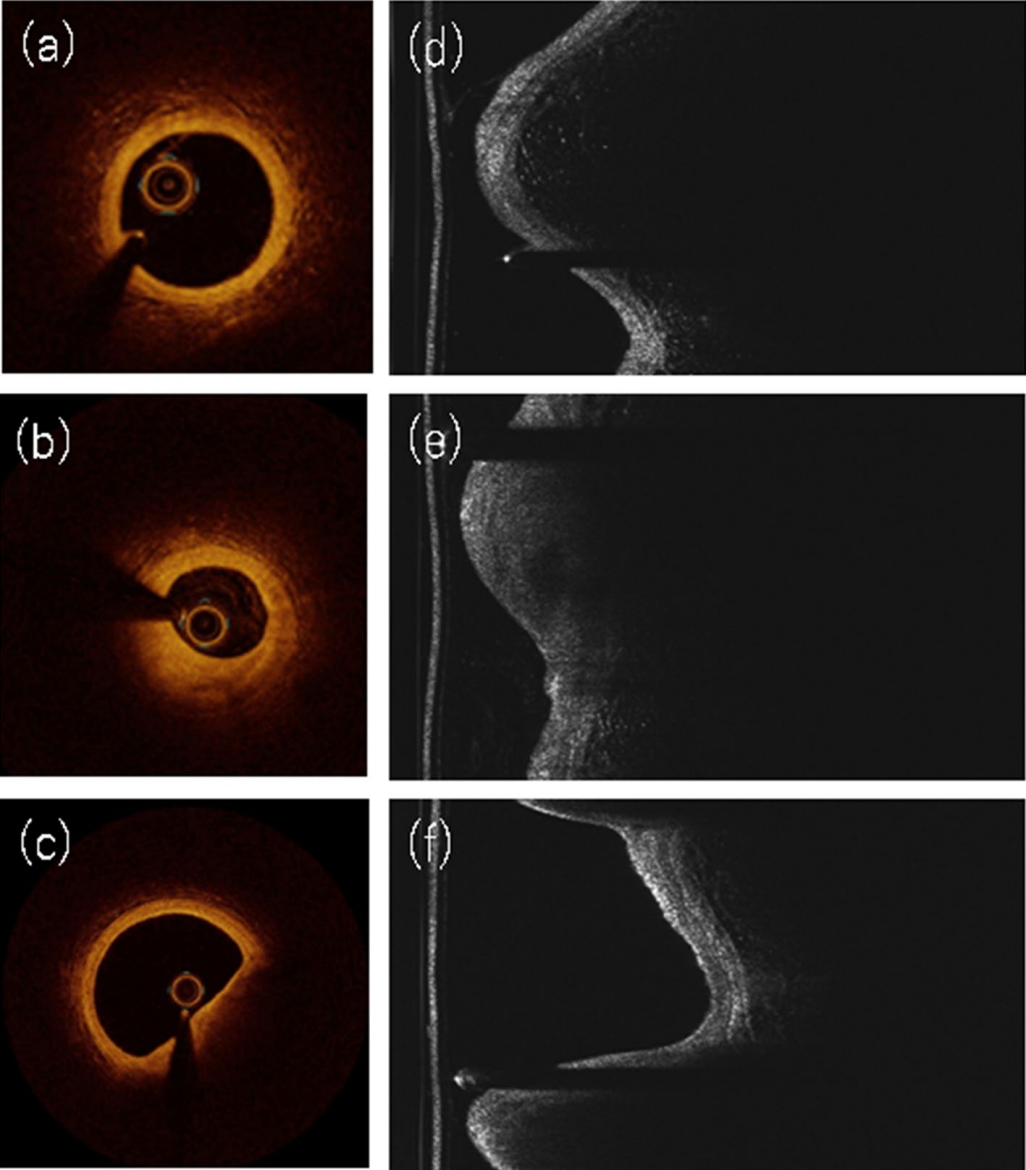
Clinical OCT images and their corresponding raw images. OCT of a normal (**a**), a stable plaque (**b**), and a vulnerable plaque (**c**) diagnosed by OCT expert cardiologists. The clinical OCT images in (**a–c**) were generated from corresponding raw data (**d–f**) by a polar coordinate transformation.

A nonculprit lesion in a CAD patient was defined as a plaque with a diameter stenosis of 25–75% on angiographic visual estimation and that was at least 10 mm away from the stented lesions, and lesions from either the culprit vessel or nonculprit vessels were included. Normal segments from non-CAD patients were selected from the proximal coronary segments with angiographically normal or minor stenosis (< 25%). The OCT images of the patient’s coronary arteries were recorded using an FD-OCT system (Dragonfly OPTIS and ILUMIEN OPTIS; Abbott Vascular, St. Paul, MN, USA) with a motorized catheter pullback system (36 mm/s). The plaque characterization of each OCT image was classified as normal, a stable plaque, or a vulnerable plaque using the previously established OCT criteria (Fig. 2)^6^. The OCT definition of a normal arterial wall was characterized by a thin (less than 300 μm) intima containing no lipid or calcification. The OCT definition of a stable plaque was characterized as the presence of intimal thickening in fibrous, fibrocalcific plaques (calcification arc ≤ 90°) or in a thick-cap (fibrous cap thickness > 100 μm) fibroatheroma. The OCT definition of a vulnerable plaque was characterized by a plaque with fibrous cap thickness < 100 μm overlying a lipid-rich plaque (lipid arc > 90°)^6^. The OCT images were labeled as being normal, a stable plaque, or a vulnerable plaque by 3 independent OCT expert cardiologists (Kume T, Soeda T, and Kubo T) who were blinded to the patient’s information. A consensus reading was obtained when there was concordance among the 3 independent readers. The interobserver reliability of the OCT diagnosis among the 3 OCT expert cardiologists was high (kappa coefficient = 0.81, 0.86, 0.97, respectively). This study allowed one lesion per patient. Therefore, if the patient had not only a normal segment but also had stable or vulnerable plaques, the stable or vulnerable plaque was assigned as the representative plaque of the patient. If the patient had more than two independent plaques with both stable and vulnerable characteristics, the vulnerable plaque was assigned as the representative plaque of the patient. If the patient had more than two independent vulnerable plaques, the lesion with the most characteristics of a vulnerable plaque was assigned as the representative plaque of the patient. The mean number of OCT frames per patient was 26 ± 16 (5.3 ± 3.3 mm).

### Development of the deep learning models

For the deep learning-based classification of the plaque characteristics, three different CNN (convolutional neural network)-based deep learning models, Inception-v3, DenseNet-121, and EfficientNet-B4, were pretrained with the ImageNet dataset^7^, and they were then trained on the prelabeled OCT images by fine-tuning. All of the models had one fully connected (FC) layer that was connected with the global average pooling, and the sizes of the FC layers were 2084, 1024, and 1280 for the Inception-v3, DenseNet-121, and EfficientNet-B4 models, respectively. The input data put into the deep learning model were resized raw OCT image data (299 × 299 × 1), and the output was the probabilities of the three types of plaques: normal, stable, and vulnerable. The details of the development protocols and the external validation are shown in the Supplemental Data.

### Testing the diagnostic capability of the AI algorithm

The diagnostic capability of the developed AI algorithm for plaque characterization was compared to the assessments made by general cardiologists in 102 patients in dataset 2. A total of 1,173 OCT frame images (normal: 425, stable plaque: 374, vulnerable plaque: 374) were randomly rearranged. The AI algorithm and four general cardiologists classified the test images as normal, a stable plaque, or a vulnerable plaque. The diagnostic accuracy was calculated as the number of true positives and true negatives using the OCT expert cardiologists’ diagnosis as the reference, and the value was divided by the total number of OCT images that were analyzed.

### The AI diagnosis and its relation to clinical outcomes

The relation between the deep learning-based plaque characterization and the long-term clinical outcomes was retrospectively evaluated in 1450 patients (dataset 3) and was compared with those based on the diagnoses made by OCT expert cardiologists. As far as the long-term clinical outcomes, two independent parameters were configured. The first one was the presence of a coronary event from the OCT-observed segments, including the need for clinically driven revascularization and the angiographic progression of CAD with a diameter stenosis > 75%. Angiographic progression of CAD was defined as lesions with less than 75% stenosis at baseline that progressed to a stenosis of 75% or more at the follow-up. Another outcome parameter was a composite of the clinical events including cardiac death, noncardiac death, and any clinically driven coronary revascularization, including ACS and a recurrence of the ischemia.

### Statistical analysis

The degree of agreement with the OCT labeling as normal, a stable plaque, or a vulnerable plaque among the 3 independent OCT expert cardiologists (interobserver variabilities) was quantified by the kappa test of concordance^8^. All of the variables were entered into a univariate analysis. Chi-squared tests were used for evaluating the categorical variables, and Student’s *t test*s were used to evaluate the continuous variables. If significant differences were recorded among the groups on the univariate analysis, a post hoc analysis using Tukey’s honestly significant difference test was used to determine the differences between the groups. The event-free data over the follow-up period were evaluated with a Kaplan–Meier analysis. Variables with a p value < 0.05 on the univariate analysis were included in the multivariate logistic regression analysis to identify the independent factors that were associated with the events from the OCT-observed segment and the composite of the clinical events. The areas under the receiver operating characteristic curves (AUCs) were used to evaluate the diagnostic ability of the model for the plaque characteristics. The AUC was calculated for one of the three classes (normal, stable, vulnerable) and for the other two classes. The AUC was also used to compare the predictive ability of events from the OCT-observed segment and the composite of the clinical events between an AI-diagnosed vulnerable plaque and an expert-diagnosed vulnerable plaque.

The mean ± SD is reported for normally distributed data. A p value < 0.05 was considered significant. Statistical analysis was performed using JMP (version 14 for Windows; SAS Institute, Cary, NC, USA).

## Results

### AI algorithm for diagnosing the OCT plaque characteristics

The classification accuracies of the three deep learning models (Inception-v3, DenseNet-121, and EfficientNet-B4) for dataset 2 were 91.2%, 92.4%, and 91.9%, respectively. The results for the AUC, Brier score, F1 score, log loss, and calibration plots for each model are shown in Supplemental Table 1 and Supplemental Fig. 4. DenseNet-121, which performed the best on these measures, was used in the following analyses. When the majority decision method (Supplemental Fig. 2) was applied to the prediction labels of dataset 2, the accuracy increased to 94.0%.

**Figure 3:**
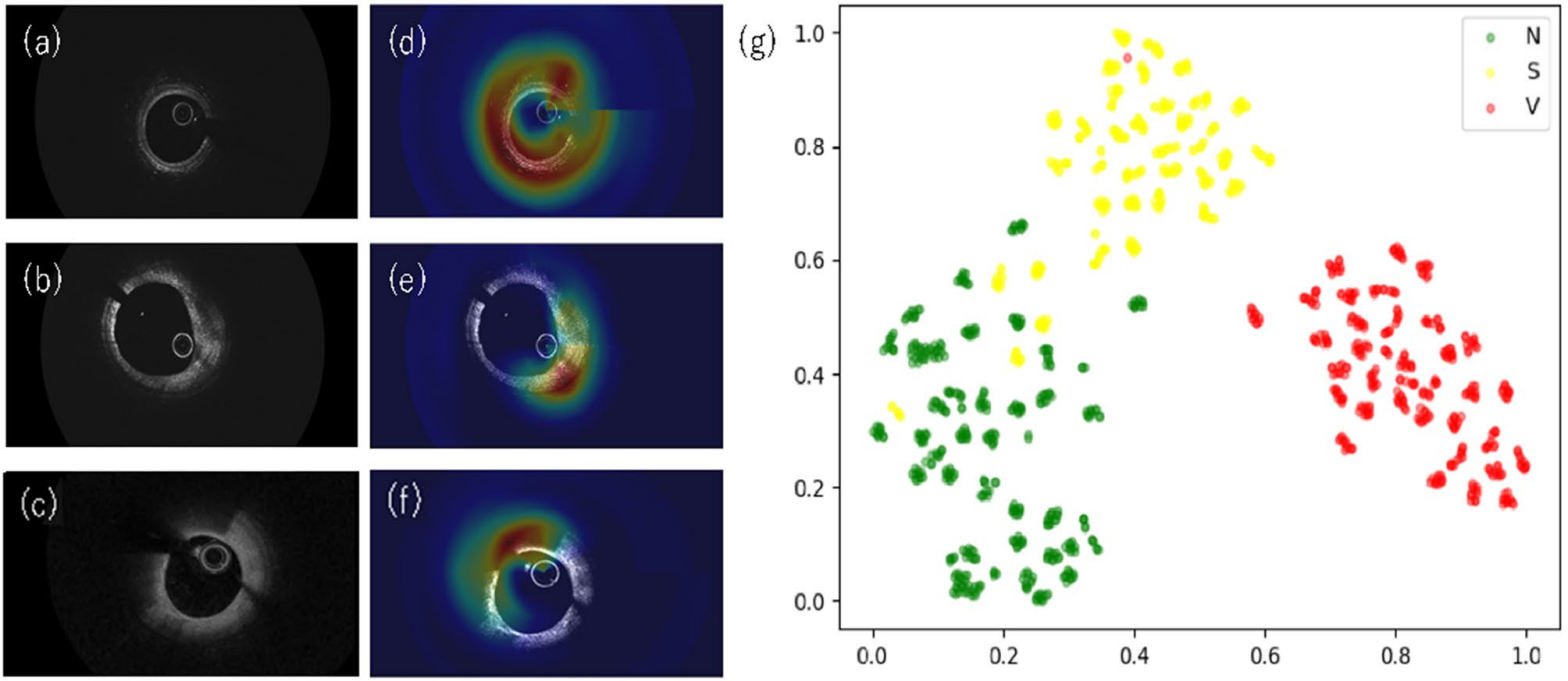
Grad-CAM analysis and t-SNE visualization of the last hidden layer for the three types of OCT imaging. Normal (**a**), stable (**b**), and vulnerable (**c**) OCT images. (**d–f**) Images of (**a–c**) overlaid with the attention map output by Grad-CAM. Expert cardiologists usually differentiate vulnerable plaque from stable plaque based on the thickness of the fibrous cap overlying the lipid component in OCT image. It is interesting to note that for stable and vulnerable plaques, the attention is on a part of the fibrous cap overlying the lipid component, whereas for normal plaques, the attention is given to the whole vessel wall in the attention map output by Grad-CAM. The high-dimensional features obtained by DenseNet-121 are dimensionally compressed by t-SNE and are represented as two-dimensional data (**g**). A total of 1,173 images of the test dataset that was obtained from 102 patients are displayed. Normal (N), stable plaque (S), and vulnerable plaque (V) images are represented as green, yellow, and red dots, respectively. The normal, stable plaque, and vulnerable plaque clusters are clearly observed. Some stable plaque data are included in the normal cluster, which is consistent with the results of the confusion matrix (Fig. 4a).

Gradient-weighted class activation mapping (Grad-CAM) was used to visualize the regions of interest for the AI algorithm in predicting a diagnosis^9^. Figure 3a–f shows the representative Grad-CAM results for each class of plaque. In the figure, the attention level is indicated with a heatmap, and the level increases from blue to red in rainbow colors. The areas in red roughly correspond to the areas that doctors focus on while making a diagnosis. Furthermore, the image distribution of dataset 2 was visualized using t-distributed stochastic neighbor embedding (t-SNE) (Fig. 3g).

### Testing of the diagnostic capability of the AI algorithm

**Figure 4:**
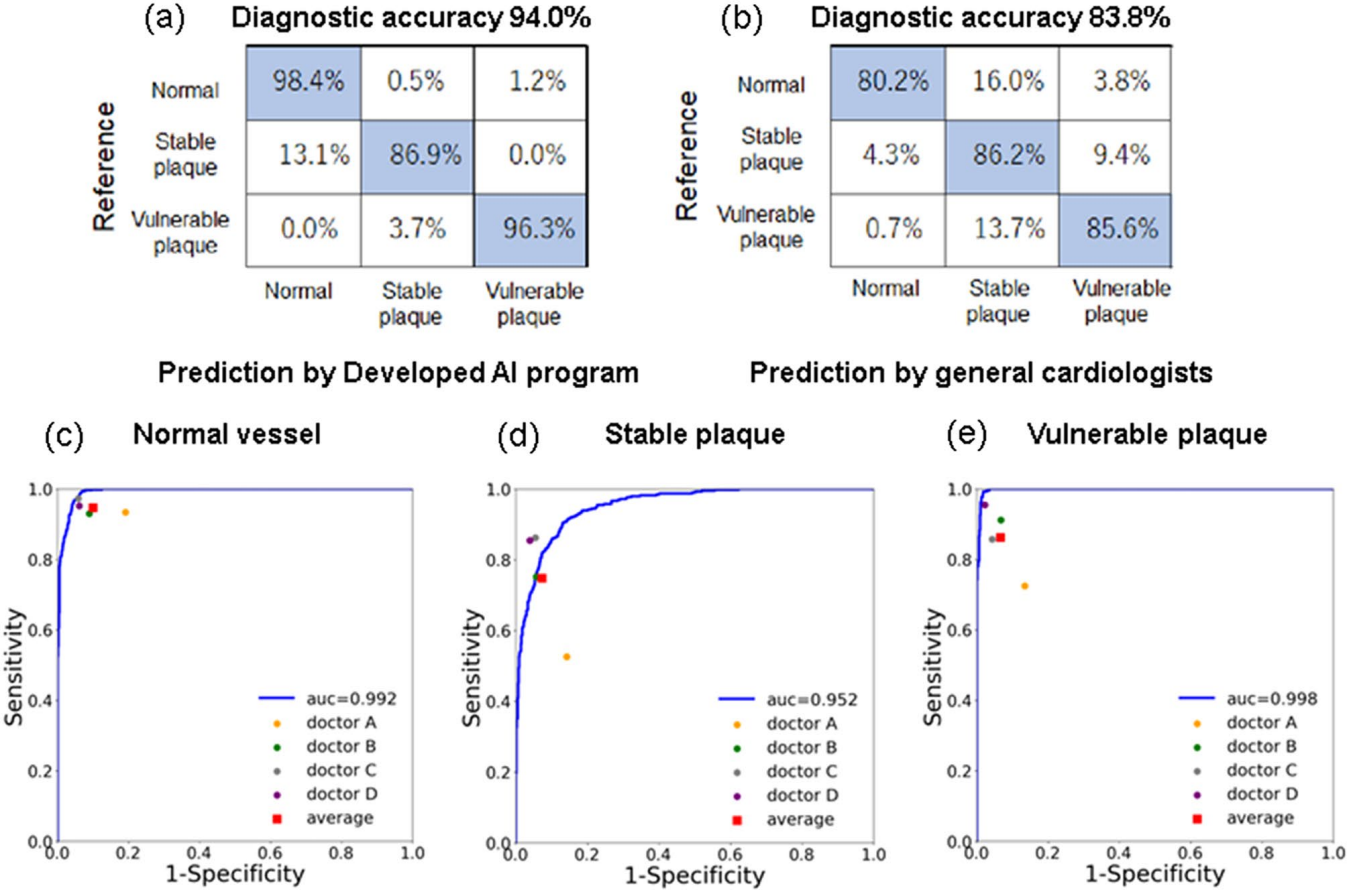
Diagnostic accuracy of the developed AI algorithm. Diagnostic accuracies of plaque vulnerability between the developed AI algorithm (**a**) and the general cardiologists (**b**) compared to the reference of the OCT-expert diagnosis. Receiver operating characteristic (ROC) curves for the AI algorithm for the differentiation of normal vessels (**c**), stable plaques (**d**), and vulnerable plaques (**e**). The dot plots represent the diagnostic accuracy of each general cardiologist.

A total of 1173 independent OCT frame images from dataset 2 were used to test the diagnostic capability of the developed AI algorithm, and the OCT images were compared with the diagnoses from the general cardiologists. The clinical profiles of dataset 2 were consistent with those of dataset 1 (age: 70.9 ± 10.9 vs. 68.0 ± 11.7 years old, male: 77.5% vs. 73.9%, respectively). Using the diagnosis made by the OCT expert cardiologists as the reference, the diagnostic accuracy of the algorithm was 94.0%, compared to an average of 83.8% among the 4 general cardiologists (Fig. 4 a,b). The individual diagnostic accuracies of the general cardiologists for plaque differentiation were 68.1%, 85.7%, 89.7%, and 91.9%, respectively. The details for the individual diagnostic accuracies of the general cardiologists are provided in Supplemental Fig. 5. The AUCs representing the ability of the AI algorithm to distinguish normal vessels, stable plaques, and vulnerable plaques were 0.992, 0.952, and 0.998, respectively (Fig. 4c–e). The time taken for plaque classification of the 1,173 OCT frame images ranged from 282 to 365 min among the general cardiologists.

### Classification of the patients with the AI algorithm

A total of 1450 patients with complete outcome data (dataset 3) were classified into three groups based on the OCT plaque diagnosis by the AI algorithm: normal (n = 435), stable plaque (n = 465), and vulnerable plaque (n = 550). In this study population, the diagnostic accuracy of the developed algorithm was 90.6% using the diagnosis made by the OCT expert cardiologists as the reference. The baseline clinical characteristics of the study population based on the plaque diagnosis by the developed algorithm are summarized in Table 1. Age, sex, hypertension, diabetes mellitus, dyslipidemia, current smoker, prior myocardial infarction, prior PCI, and the index clinical presentation were significantly different among the three groups. The age of patients with vulnerable plaques was significantly higher than that of patients with a normal OCT registered lesion. The estimated glomerular filtration rate (eGFR), LDL-Cho, HDL-Cho, and the medications at baseline were significantly different among the three groups. Patients with vulnerable plaques had higher levels of LDL-Cho and lower levels of HDL-Cho than the patients with a normal OCT registered segment. These clinical characteristics were similar to those diagnosed by OCT expert cardiologists (Supplemental Table 2). The analyzed lesions were identified in the left anterior descending artery (49.3%), in the left circumflex artery (18.2%), in the right coronary artery (31.0%), and in the left main trunk (1.5%).

**Table 1:** Clinical characteristics of the patients in dataset 3 according to the classification by the AI algorithm.

	Overall (n = 1450)	Vulnerable (n = 550)	Stable (n = 465)	Normal (n = 435)	p value
Age (years)	68.0 ± 11.3	69.0 ± 10.8*	67.8 ± 10.8	66.9 ± 12.3	0.022
Male	1081	412	365	304	0.012
Body mass index (kg/m^2^)	23.9 ± 3.7	23.7 ± 3.7	24.1 ± 3.6	24.0 ± 3.9	0.260
Hypertension	1071	422	361	288	< 0.001
Diabetes mellitus	544	223	184	137	0.006
Dyslipidemia	1029	377	351	301	0.042
Current smoker	349	160	95	94	0.014
Prior myocardial infarction	428	127	168	133	< 0.001
Prior PCI	671	173	286	212	< 0.001
**Index clinical presentation**					< 0.001
Acute coronary syndrome	495	353	87	55	
Chronic coronary artery disease	644	152	281	211	
Others	311	45	97	169	
**Laboratory data**					
Serum creatinine (mg/dL)	1.17 ± 1.55	1.28 ± 1.73	1.15 ± 1.40	1.08 ± 1.45	0.121
eGFR (mL/min/1.73 m^2^)	66.0 ± 23.5	65.1 ± 25.6	64.4 ± 21.4^†^	68.6 ± 22.4	0.025
HbA1c (%)	6.35 ± 1.11	6.45 ± 1.28*	6.34 ± 0.96	6.26 ± 1.07	0.060
LDL-Cho (mg/dL)	96.5 ± 31.1	104.3 ± 34.2*^‡^	88.4 ± 28.8^†^	96.4 ± 30.0	< 0.001
HDL-Cho (mg/dL)	48.1 ± 13.0	46.9 ± 12.1*	47.9 ± 12.6	49.5 ± 14.4	0.029
Triglycerides (mg/dL)	140 ± 87	138 ± 91	146 ± 90	135 ± 78	0.233
Uric acid (mg/dL)	5.66 ± 1.40	5.65 ± 1.41	5.76 ± 1.38	5.56 ± 1.39	0.136
C-reactive protein (mg/dL)	0.51 ± 1.58	0.54 ± 1.34	0.52 ± 1.72	0.46 ± 1.71	0.749
BNP (pg/mL)	146 ± 348	162 ± 280	114 ± 188	164 ± 506	0.131
**Medications at baseline**					
Antiplatelet therapy	1183	430	388	365	< 0.001
Statins	820	245	314	261	< 0.001
Beta-blockers	487	146	198	143	< 0.001
ACEI/ARB	742	265	266	211	< 0.007

The values are presented as the means ± SD.

*p < 0.05 vulnerable vs. normal.

^†^p < 0.05 stable vs. normal.

^‡^p < 0.05 vulnerable vs. stable.

*PCI* percutaneous coronary intervention, *eGFR* estimated glomerular filtration rate, *HbA1c* hemoglobin A1c, *LDL-Cho* low-density lipoprotein cholesterol, *HDL-Cho* high-density lipoprotein-cholesterol, *BNP* brain natriuretic peptide, *ACEI* angiotensin-converting enzyme inhibitor, *ARB* angiotensin II receptor blocker.

### The relation between AI diagnosis of plaque vulnerability and clinical outcomes

The median duration between the index OCT examination and the determination of the clinical outcome was 530 days **(**interquartile range 310–1105 days**)**.

**Figure 5:**
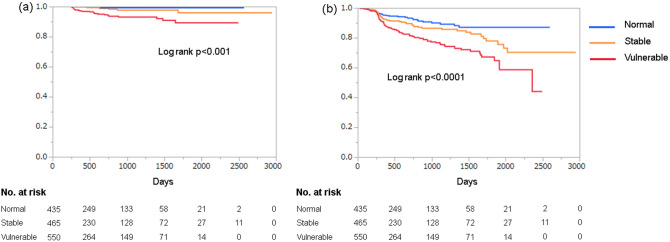
Clinical outcomes in patients with CAD diagnosed by the AI algorithm. Kaplan–Meier curves of the event-free survival from the OCT-observed segments (**a**) and the composite of the clinical events (**b**) according to classification by the AI algorithm. Patients with OCT-diagnosed vulnerable plaques showed higher cumulative rates for both endpoints than the patients with OCT-diagnosed normal and stable plaques.

The numbers of OCT-observed segment events and the composite of the clinical events were 31 and 187, respectively. The 31 events from the OCT-observed segments consisted of clinically driven coronary revascularization (n = 18: 3 ACS and 15 recurrent angina pectoris) and angiographic progression (diameter stenosis > 75%) of the predetermined segment (n = 13). The Kaplan–Meier analysis showed that the patients with AI-diagnosed vulnerable plaques had significantly higher cumulative rates of events from the OCT-observed segments than the patients with AI-diagnosed normal and stable plaques (Fig. 5a). Sixteen lesion-related events occurred in the patients with AI-diagnosed vulnerable plaques, whereas two events occurred in patients with AI-diagnosed stable plaques and normal plaques during the follow-up period. On the multivariate logistic regression analyses, only the AI diagnosis of an OCT vulnerable plaque was independently associated with the events from the OCT-observed segments (p < 0.001) (Table 2).

**Table 2:** Events from the OCT-observed segments and the composite of clinical events.

	Events from the OCT-observed segments (n = 31)			Composite of the clinical events (n = 187)		
	Odds ratio	95% CI	p value	Odds ratio 95% CI p value	95% CI	p value
Age	0.983	0.940–1.028	0.450	1.013	0.992–1.034	0.225
Male	3.057	0.651–14.347	0.157	1.353	0.802–2.282	0.257
Hypertension	0.915	0.332–2.523	0.864	0.923	0.558–1.524	0.751
Diabetes mellitus	1.350	0.563–3.238	0.502	1.221	0.810–1.842	0.340
Dyslipidemia	1.475	0.416–5.226	0.547	1.006	0.582–1.740	0.982
Current smoker	0.729	0.235–2.268	0.586	0.798	0.464–1.373	0.416
Prior myocardial infarction	1.053	0.308–3.600	0.934	0.750	0.436–1.292	0.301
Prior PCI	1.163	0.314–4.312	0.821	1.337	0.756–2.361	0.318
Index clinical presentation: acute coronary syndrome	0.637	0.203–1.996	0.439	1.228	0.750–2.012	0.414
eGFR	0.983	0.966–1.001	0.062	0.991	0.982–1.000	0.047
LDL-Cho	1.008	0.994–1.023	0.253	1.003	0.996–1.010	0.442
HDL-Cho	0.984	0.944–1.026	0.444	0.989	0.972–1.006	0.198
Antiplatelet therapy	0.727	0.198–0.673	0.631	0.896	0.477–1.683	0.733
Statins	0.706	0.217–2.299	0.563	0.892	0.512–1.554	0.688
Beta-blockers	2.087	0.718–6.063	0.177	1.023	0.638–1.650	0.916
ACEI/ARB	0.551	0.201–1.509	0.245	0.874	0.552–1.384	0.567
Plaque characteristics: vulnerable plaque	13.526	3.730–49.051	< 0.001	2.295	1.478–3.562	< 0.001

*CI* confidence interval, *PCI* percutaneous coronary intervention, *eGFR* estimated glomerular filtration rate, *LDL-Cho* low-density lipoprotein cholesterol, *HDL-Cho* high-density lipoprotein-cholesterol, *ACEI* angiotensin-converting enzyme inhibitor, *ARB* angiotensin II receptor blocker.

Furthermore, the patients with AI-diagnosed vulnerable plaques had significantly higher cumulative rates for the composite clinical outcomes than the patients with AI-diagnosed normal and stable plaques (Fig. 5b), and AI-vulnerable plaques and eGFR were independently associated with the composite of the clinical events (p = 0.047 and p < 0.001, respectively) (Table 2).

The Kaplan–Meier analyses that were obtained by the OCT diagnosis of the AI algorithm were comparable to those diagnosed by the OCT expert cardiologists (Supplemental Fig. 6). The AUC value of the AI vulnerability for events from the OCT-observed segments was 0.719, indicating a moderate accuracy, and this accuracy (0.742) was comparable to the AUC of the OCT expert-vulnerable plaques. In addition, the AUCs of the AI vulnerability and the expert vulnerability for the composite clinical events were 0.590 and 0.592, respectively.

## Discussion

The salient results of the present study are as follows: (1) the deep learning-based AI algorithm that was developed in this study demonstrated excellent differentiation among normal, stable plaques, and vulnerable plaques, and its diagnostic capability was better than that of general cardiologists; and (2) CAD patients with AI-classified vulnerable plaques had significantly higher rates of coronary lesion-related and clinical events than patients with AI-classified normal coronary arteries and stable plaques.

Patients with CAD have a high risk for recurrent cardiovascular events, not only in lesions that were previously treated with PCI, but also from untreated, nonculprit lesion sites^1,2^. For lesions that were previously treated with PCI, improvements in the drug-eluting stents and antiplatelet therapy have significantly decreased the risk for PCI-related secondary events^10^. On the other hand, the rate of cardiovascular events from untreated nonculprit lesions remains very high, despite intensive strategies for risk modification. Accordingly, the effective identification of patients who are at a high risk of developing events from nonculprit lesions is crucial regarding the long-term care of CAD patients. OCT is a high-resolution intravascular imaging technique that uses near-infrared spectroscopy, and it can differentiate plaque instability in vivo^4,5^. However, the routine analysis of coronary lesions with OCT has practical limitations because of the large numbers of OCT images that need to be evaluated, and OCT image interpretation requires substantial experience. To address this practical issue, we sought to develop an AI algorithm, as an accurate and automatic diagnostic tool, for the vulnerability of nonculprit coronary plaques and evaluate the relation between AI diagnosis of plaque vulnerability and clinical outcomes in patients with CAD.

Various medical image analyses using deep learning have been studied as diagnostic aids for clinicians, and these imaging modalities include CT images, retinal fundic images, pathological images, mammography images, and skin images^11,12^. Recently, Liu and colleagues reported the application of an AI program to OCT for coronary plaque characterization^13^. Based on their relatively small dataset, they could detect vulnerable plaques with a good diagnostic accuracy (detection quality score 88.46%) using cross-sectional images (generated by a polar coordinate transformation) of the vessels. More recently, Min and colleagues reported the application of an AI program to OCT for identifying thin-cap fibroatheroma using 602 coronary lesions from 602 angina patients and a developed AI program could accurately detect a thin-cap fibroatheroma (AUC = 0.96)^14^. The present study used raw OCT data instead of cross-sectional transformation images. Although this process is not intuitive for humans, it is thought that information loss occurs due to the polar transformation. After a polar coordinate transformation, the information in an image is sparser in the outer part of the image than in the center. Therefore, the information loss is more significant in the outer part of the image. This can be complemented by bilinear or bicubic processing, but these techniques are not sufficient. When DenseNet-121 was trained using the polar-transformed dataset, the accuracy for the test data (dataset 2) was 89.5%, which was 2.9 percentage points lower in accuracy than when using the raw data. In addition, by using a graph convolutional neural network (GCN), it is possible to use the raw data of OCT images as the polar coordinate data without any information loss. This is a promising method for the future. However, the OCT polar coordinate image used in the present study had 488,064 nodes (984 × 496) in the raw data and 89,401 nodes (299 × 299) in the resized images for input into the CNN model, which is a very large data size for GCN computation and requires a high computational cost for training. Another notable feature of this study was that OCT images were prelabeled based on the consensus of three OCT experts. According to such a novel approach to training, the AI algorithm demonstrated a very high diagnostic capability (92.4%), and this diagnostic rate was better than that of that from general cardiologists. The time between inputting a single OCT image into DenseNet-121 and obtaining the result was 35 ms; of course, with a more powerful GPU, it could be even faster. Such technology could support clinicians in decision-making and can reduce the burden of patient care. In addition, since this is a very short amount of time, there are potential applications for determining the plaque vulnerability by using a deep learning analysis of angiographic images in real time during coronary angiography and PCI.

In this study, the relation between AI diagnosis of plaque vulnerability and clinical outcomes of CAD patients was also tested. To the best of our knowledge, no previous study has used a well-defined cohort of OCT image samples with a well-established large clinical database to assess the clinical implications of medical AI with respect to its relation to future cardiac events. Several studies have attempted to use intravascular imaging for prognostic stratification. The PROSPECT I and II, AtheroRemo-IVUS, and LRP studies have shown the ability of grayscale IVUS, radiofrequency IVUS, and near-infrared spectroscopy IVUS to predict events in patients presenting with ACS and in stable patients undergoing an index PCI^1,2,15,16^. These IVUS studies have demonstrated the importance of identifying high-risk lesions in patients with ischemic heart disease. We recently showed that vulnerable plaques that were diagnosed with OCT were associated with the subsequent progression of coronary stenosis, as well as future major adverse cardiac events^4,5^. In the present study, CAD patients with AI-diagnosed OCT vulnerable plaques had significantly worse lesion-specific outcomes. Furthermore, the presence of AI-diagnosed vulnerable plaques was also associated with a higher incidence of composite clinical outcomes, including noncardiac death, in CAD patients. The reason for this finding is unclear, but the comorbidities of CAD patients, such as chronic inflammatory diseases and malignant tumors, are known to correlate with enhanced plaque vulnerabilities in coronary arteries^17^. However, the prognostic value of AI vulnerability for the lesion-specific events was higher (AUC = 0.719) than that for the composite of the clinical events (AUC = 0.590). These data suggested that AI diagnosis of plaque vulnerability might be more useful for evaluating the prognostic value form the OCT-observed segments rather than the composite of the clinical events including cardiac death, noncardiac death and any clinically driven coronary revascularization.

In the present study, patients with vulnerable plaques diagnosed by AI algorithm had a large number of coronary risk factors suggesting that this new technology could differentiate high-risk patients who should receive intensive medical management without the need for expertise and experience with OCT. Furthermore, we will be able to adapt this new technology to noninvasive imaging modalities, including coronary computed tomography and magnetic resonance imaging, for predicting cardiovascular events in primary care settings in the future. In addition, the automatic diagnosis of the plaque vulnerability by an AI program will provide useful information not only for the prognostic stratification but also for planning the PCI treatment strategies for patients in the cardiac catheterization laboratory. The plaque characteristics of the stent edge landing zone may affect the stent length selection. Interventional cardiologists prefer to position the edges of the stents into reference segments that are normal or that are at least less diseased segments. A stent landing edge should be avoided in segments with a vulnerable plaque even if they have an angiographically normal appearance because of the possible development of stent edge dissection and stent edge restenosis. Therefore, a quick diagnosis of the plaque vulnerability by an AI program is very useful in the cardiac catheterization laboratory when interventional cardiologists select the stent edge landing zone. From this perspective, integration of an AI program in an OCT imaging apparatus might automatically and efficiently promote the practice of PCI and provide risk stratification of individual CAD patients. Furthermore, the automatic determination and localization of vulnerable plaques with an AI program might help us change the PCI strategy, as well as the medical treatment, in individual CAD patients.

### Limitations

Several limitations of the present study should be addressed. First, this was a retrospective cohort analysis, which may have selection bias. In addition, information about the clinical characteristics, including the lipid profiles, at the follow-up was lacking. However, a large number of CAD patients with complete baseline clinical characteristics were recruited. Second, OCT images with severe calcification (calcification arc > 90°) were excluded from this study because the impact of severe calcification detected by OCT in de novo lesions on clinical outcomes remains controversial. Third, lesions with OCT images that showed a fresh thrombus and/or plaque rupture indicating culprit lesions were excluded from this study. However, all of the registered lesions were not functionally tested using a pressure guidewire. Therefore, coronary angiograms have limitations in determining whether a lesion is culprit or nonculprit. Fourth, to test the diagnostic capability of the AI program, four general cardiologists who had different years of experience were recruited. Shibutani et al. recently evaluated the effect of the observers’ years of experience on the interpretation of OCT images with reference to the histopathological findings^18^. They reported that the interpretation ability of OCT varied significantly among observers, and it is possible that there was a significant selection bias of the observers in the present study. Fifth, the prognosis value of the AI vulnerability for the clinical outcomes in CAD patients was not actually higher than that of the OCT expert vulnerability (AUC of AI vulnerability: 0.590, AUC of expert vulnerability: 0.592). However, the automatic analysis of numerous OCT images by AI-assisted computer systems without the input by expert cardiologists may be able to substantially distinguish patients who are at a high risk without additional workload to the medical staff and hospital resources. In this study, the predictive capability of the algorithm on the clinical outcomes was not validated in an external independent patient cohort, so a future prospective study is needed to confirm the present findings. Last, the clinical event rates, especially the incidence of acute coronary syndrome, were low in the present study.

## Conclusions

An AI program for analyzing intracoronary OCT imaging can assist cardiologists in diagnosing coronary plaque vulnerability and identifying CAD patients with a high probability for the development of future cardiovascular events. The clinical application of an AI system could reduce the medical workload and promote the individualized care of CAD patients based on its prognostic value of clinical outcomes.

## Supplementary Information

Supplementary Information.

## Data Availability

All data generated or analyzed during this study are available from the corresponding author on reasonable request.
