# Supplementary material for: Automated diagnosis of optical coherence tomography imaging on plaque vulnerability and its relation to clinical outcomes in coronary artery disease
Source: Sci Rep. 2022 Aug 18;12:14067. doi: 10.1038/s41598-022-18473-5 (PMC9388661; doi:10.1038/s41598-022-18473-5)

**Supplemental Appendix**

**Automated diagnosis of optical coherence tomography imaging on plaque vulnerability and its relation to clinical outcomes in coronary artery disease**

These data are intended for publication as an online data supplement.

Supplemental Method (Deep-learning procedures)

Figure Legends for Supplemental Figures

2 Supplemental Tables

6 Supplemental Figures

**Supplemental Methods**

**Training of the deep learning models**

Among the deep learning models, convolutional neural network (CNN)-based models show high accuracy in image recognition. CNN-based models have been widely used not only in the field of general image recognition but also in the field of medical image diagnosis, and their high recognition accuracy has been reported. The ImageNet dataset is a teacher-labeled dataset of 1000 categories and consists of more than 14 million general images. CNN-based image recognition models can achieve a high accuracy with fewer images and can shorten the training time by pretraining with the ImageNet dataset. This method is commonly used in the field of medical image recognition.

A total of 44,947 OCT frame images, including 17,548 normal, 16,115 stable plaque, and 11,284 vulnerable plaque OCT images derived from 1,689 patients (patients with normal coronaries: 572, stable plaque: 546, vulnerable plaque: 571) (dataset 1), were used. Each OCT image was randomly assigned to the training or validation dataset (the ratio was 8:2). A single patient’s data was not split into training and validation data because the adjacent images in a single patient’s data are similar, and splitting similar images into training and validation datasets would result in an unfairly high accuracy. In addition, the raw OCT image was used as the input data before it was transformed into the cross-sectional image of the coronary artery, as shown in **Figure 2**. Since the image size of the raw data was 984×496 pixels, it was reshaped and was converted to an image size of 299×299 pixels (**Supplemental Figure 1**). The training was conducted in 20 epochs, and the weights when the loss was lowest for the validation dataset were used to predict the labels of the test dataset. As the loss function, categorical cross-entropy was used. The optimizer was adamW (learning rate = 0.0001, weight decay = 0.0001) ^1^. The last hidden layer representation of DenseNet-121 was compressed to two-dimensional data by t-distributed stochastic neighbor embedding (t-SNE). This is a nonlinear dimensionality reduction algorithm that can compress high-dimensional data into low dimensions, such as in two or three dimensions. Therefore, it enables us to intuitively understand the image data distribution.

**Data labeling for the deep learning AI analysis**

To increase the accuracy, a majority decision method was introduced (**Supplemental Figure 2**) ^2^. Since the plaque exists within a specific range in the coronary artery, the same teacher label was naturally assigned to multiple consecutive OCT images. However, sparse prediction errors, such as the predicted label "stable plaque" in **Supplemental Figure 2**, occur even when the AI’s prediction accuracy is over 90%. This prediction error is corrected by a majority decision of the surrounding predicted labels. As shown in **Supplemental Figure 2**, the prediction label was corrected while scanning a window with a window size of 5. Although the prediction labels of the OCT expert cardiologists were only given to a certain successive OCT image region, the AI predictions were also made for the images outside of that region. Therefore, a majority correction was performed on all of the teacher-labeled regions.

The following rules were applied to classify a patient by plaque prediction with the AI algorithm. If all of the images were predicted to be normal for one patient’s OCT data, the patient was given a normal label. When even one image was predicted to be a stable plaque from the above case, the patient was given a diagnostic label of a stable plaque. If there was a case in which even one image was predicted to be a vulnerable plaque, the patient was given a diagnostic label of a vulnerable plaque.

For the Kaplan–Meier analysis, 10-fold nested cross-validations were adopted to make label predictions on all of the data. The advantage of using 10-fold nested cross-validation is that all samples are labeled as test data for prediction, and the use of an ensemble of multiple trained models in the test data prediction results in high accuracy. When using the hold-out method for data split, the training data, validation data, and test data are split in a ratio of 8:1:1 or 6:2:2, for example. Deep learning models are trained using training data, parameters are tuned to achieve high performance on validation data, and then predictions are made on test data. Thus, only 10% or 20% of the total data is treated as test data. In the case of N-fold cross-validation, the data is divided into N folds, N−1 folds of which are training data and another one fold is validation data. The deep learning model is trained N times while changing the fold selected as the validation data, and the model is evaluated by the average value of the accuracy against the validation data. However, since test data is not used, label prediction cannot be performed on unknown data (test data) after tuning parameters with training and validation data. The holdout and ordinary k-fold cross-validation methods treat a part of the total data as a test dataset. In contrast, with a k-fold nested cross-validation, all of the data can be treated as the test dataset, and prediction labels can be applied to them through repeated training. **Supplemental Figure 3** shows how to divide the data in a 10-fold nested cross-validation.

For all of the calculations using deep learning, a custom-made PC with a CPU (Intel Xeon(R) W-2123, Intel Corporation, Santa Clara, USA) and a GPU (TITAN RTX, 24 GB, NVIDIA Corporation, Santa Clara, USA) was used. The installed OS was Ubuntu 18.04 LTS. PyTorch 1.50 Torchvision 0.6.0 (<https://github.com/pytorch/vision>) was used to build the deep learning models.

**References**

1. Ilya F, Hutter F. Decoupled weight decay regularization. *Proceedings of the International Conference on Learning Representations (ICLR)*  [arXiv. **1711**:05101](https://arxiv.org/abs/1711.05101) (2019).

2. Matsumoto T. et al. Deep-UV excitation fluorescence microscopy for detection of lymph node metastasis using deep neural network. *Sci Rep.* **9**:16912 (2019).

**Supplemental Figure Legends**

**Supplemental Figure 1.** Layout of a deep learning model’s prediction labels on an OCT image input

The OCT raw image data were resized to a pixel size of 299×299 and were input to the deep learning model. They were then output as a three-class prediction, which is a three-dimensional vector representing the probability of each class. The sum of the probabilities is 1, and the class with the highest probability is the class that is predicted by the deep learning model.

**Supplemental Figure 2**. Schematic image of the majority decision process

An image predicted as a stable plaque (S) exists between the images predicted as a vulnerable plaque (V). The predicted label of a stable plaque is converted to a label of a vulnerable plaque after the majority decision. The window size is 5, which was scanned and adapted to all predicted labels.

**Supplemental Figure 3.** The 10-fold nested cross-validation and ensemble averaging of the multiple models

A total of 44,947 images (dataset 1) were divided into 10 partitions. For the same reason as in the “Training deep learning models” subsection, the data of a single patient were treated as a single set and were not split into training, validation, and test data. Since all of the combinations were trained, a total of 90 DenseNet-121 models were trained individually. The weights of the lowest loss for the validation dataset were used to predict the labels of the test dataset. For a single test datum, nine prediction labels were given by the nine models. The nine models were ensembled to further increase the accuracy, in which the average of the output vectors from the models was taken for the final prediction labeling.

**Supplemental Figure 4.** Calibration plots of each deep learning model using the test data (dataset 2)

Horizontal axis of the calibration plot: This is the predicted probability for the class where the objective variable (teacher label) is 1 when a certain data point is input into the deep learning model. The horizontal axis is delimited by multiple bins. The vertical axis of the calibration plot: the percentage of data for which the objective variable is 1 in each bin. This is a plot to check whether the predicted probability and the percentage of data, where the objective variable is 1, actually match. When the predicted probability and the proportion match, the calibration plot has a slope of 45 degrees. Therefore, the closer to the dotted line in the figure, the better the prediction performance of the model.

**Supplemental Figure 5.** Diagnostic accuracy of each general cardiologist compared to the diagnosis made by OCT expert cardiologists, which was used as the reference

The individual diagnostic accuracies of the general cardiologists (experience of cardiac catheterization: doctor a: 1 year, doctor b: 2 years, doctor c: 3 years, doctor d: 4 years) for plaque differentiation were 68.1%, 85.7%, 89.7%, and 91.9%, respectively (a-d).

**Supplemental Figure 6.** Prediction of the clinical outcomes in patients with CAD by OCT expert cardiologists

Kaplan–Meier curves of the OCT-observed segment event-free survival curve (a) and the composite of the clinical event-free survival curve (b) according to the classification by OCT expert cardiologists. Patients with OCT-diagnosed vulnerable plaques had higher cumulative rates of both endpoints than the patients with OCT-diagnosed normal and stable plaques.

**Supplemental Table 1.** Comparison of each CNN model using the evaluation indices

| Model | AUC | Brier score | Log loss | F1 |
| --- | --- | --- | --- | --- |
| Inception-v3 | 0.972 | 0.041 | 0.214 | 0.916 |
| DenseNet-121 | 0.981 | 0.032 | 0.202 | 0.931 |
| EfficientNet-B4 | 0.977 | 0.038 | 0.209 | 0.924 |

CNN: convolutional neural network, AUC: area under the curve

Brier Score (BS) is the mean squared difference between the true class and the predicted probability. It is defined by the following equation in the case of multi-class classification.

$$BS= \frac{1}{2N}\sum_{i=1}^{N} {\sum_{k=1}^{K} (C_{ik}-p_{ik})}^{2}$$

where $N$ is the number of samples, $K$ is the number of classes, $C_{ik}$ is the teacher label of class $k$ for sample $i$, and $p_{ik}$ is the predicted probability for sample $i$ belonging to class $k$. When BS is close to 0, it indicates high prediction accuracy.

**Supplemental Table 2.** Clinical characteristics of the patients in dataset 3 according to the classifications by the OCT expert cardiologists

|  | Overall  (n=1450) | Vulnerable  (n=482) | Stable  (n=485) | Normal  (n=483) | p value |
| --- | --- | --- | --- | --- | --- |
| Age (y) | 68.0±11.3 | 68.9±10.6* | 68.1±10.8 | 67.1±12.3 | 0.049 |
| Male | 1081 | 364 | 373 | 344 | 0.106 |
| Body mass index (kg/m^2^) | 23.9±3.7 | 23.7±3.7 | 24.0±3.6 | 24.0±3.9 | 0.376 |
| Hypertension | 1071 | 366 | 386 | 319 | <0.001 |
| Diabetes mellitus | 544 | 195 | 203 | 146 | <0.001 |
| Dyslipidemia | 1029 | 334 | 362 | 333 | 0.122 |
| Current smoker | 349 | 139 | 101 | 109 | 0.014 |
| Prior myocardial infarction | 428 | 106 | 175 | 147 | <0.001 |
| Prior PCI | 709 | 158 | 309 | 242 | <0.001 |
| Index clinical presentation |  |  |  |  | <0.001 |
| Acute coronary syndrome | 495 | 349 | 88 | 58 |  |
| Chronic coronary artery disease | 644 | 133 | 397 | 245 |  |
| Others | 180 |  |  | 180 |  |
| Laboratory data |  |  |  |  |  |
| Serum creatinine (mg/dL) | 1.17±1.55 | 1.26±1.73 | 1.22±1.57 | 1.05±1.32 | 0.089 |
| eGFR (mL/min/1.73 m^2^) | 66.0±23.5 | 65.6±25.3 | 63.9±23.0^†^ | 68.3±21.8 | 0.023 |
| HbA1c (%) | 6.35±1.11 | 6.45±1.28 | 6.36±0.99 | 6.25±1.06 | 0.063 |
| LDL-Cho (mg/dL) | 96.5±31.8 | 107.0±34.3*^‡^ | 87.6±28.3^†^ | 95.9±29.9 | <0.001 |
| HDL-Cho (mg/dL) | 48.1±13.1 | 46.8±11.9* | 47.7±12.5 | 49.6±14.5 | 0.016 |
| Triglycerides (mg/dL) | 149±87 | 139±94 | 146±90 | 135±77 | 0.187 |
| Uric acid (mg/dL) | 5.65±1.40 | 5.64±1.37 | 5.73±1.45 | 5.58±1.40 | 0.275 |
| C-reactive protein (mg/dL) | 0.51±1.58 | 0.57±1.40 | 0.47±1.57 | 0.48±1.75 | 0.574 |
| BNP (pg/mL) | 146±348 | 163±284 | 118±196 | 160±489 | 0.204 |
| Medications at baseline |  |  |  |  |  |
| Antiplatelet therapy | 1183 | 381 | 393 | 409 | <0.001 |
| Statins | 820 | 205 | 324 | 291 | <0.001 |
| Beta-blockers | 487 | 121 | 207 | 159 | <0.001 |
| ACEI/ARB | 742 | 221 | 291 | 230 | <0.001 |

The values are presented as the means ± SD. *p<0.05 vulnerable vs. normal. ^†^p<0.05 stable vs. normal. ^‡^p<0.05 vulnerable vs. stable. PCI: percutaneous coronary intervention, eGFR: estimated glomerular filtration rate, HbA1c: hemoglobin A1c, LDL-Cho: low-density lipoprotein cholesterol, HDL-Cho: high-density lipoprotein-cholesterol, BNP: brain natriuretic peptide, ACEI: angiotensin-converting enzyme inhibitor, ARB: angiotensin II receptor blocker.

**Supplemental Figure 1**


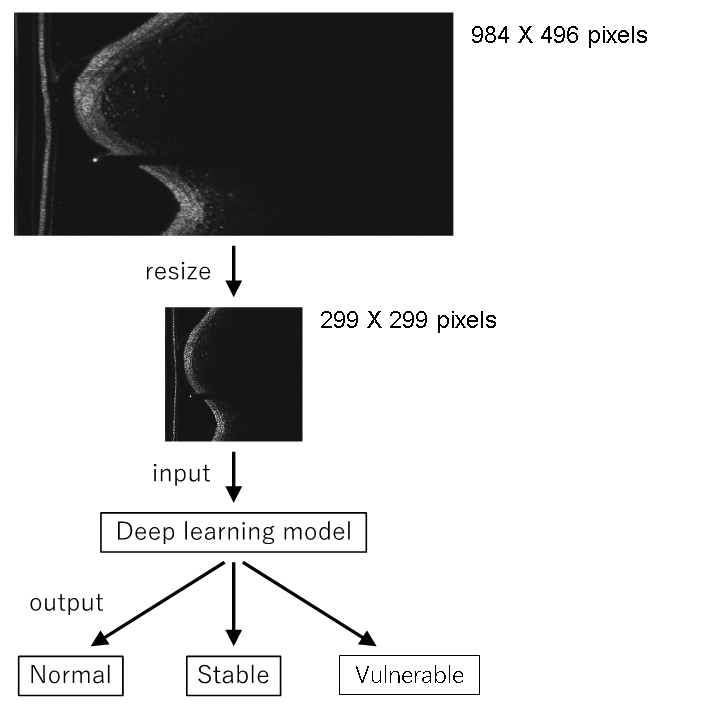


**Supplemental Figure 2**


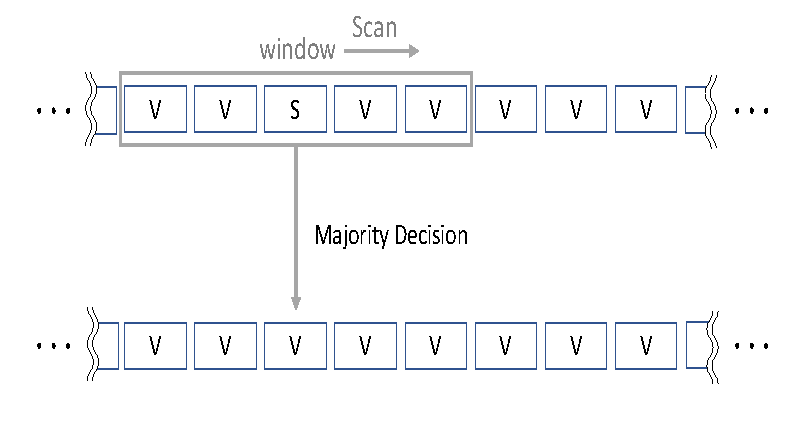


**Supplemental Figure 3**


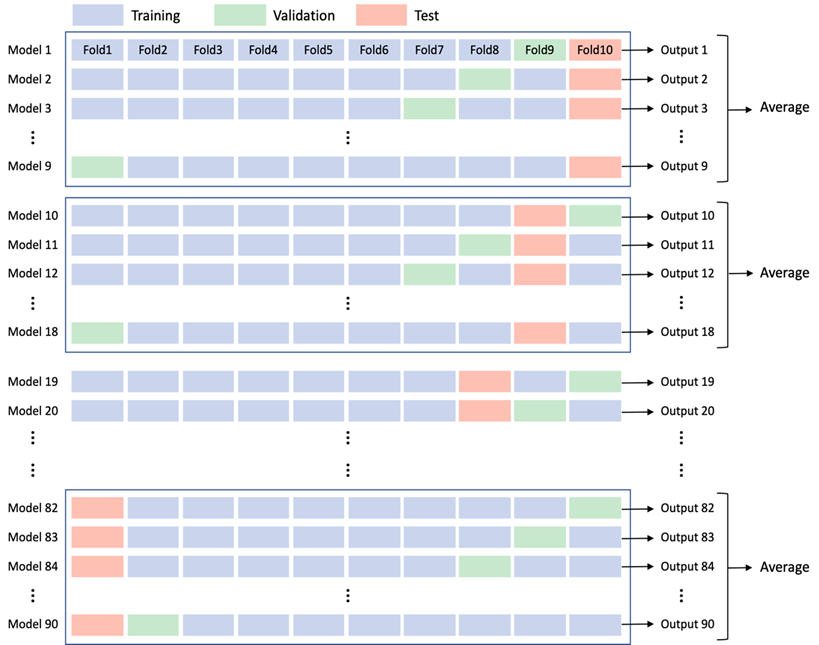


**Supplemental Figure 4**


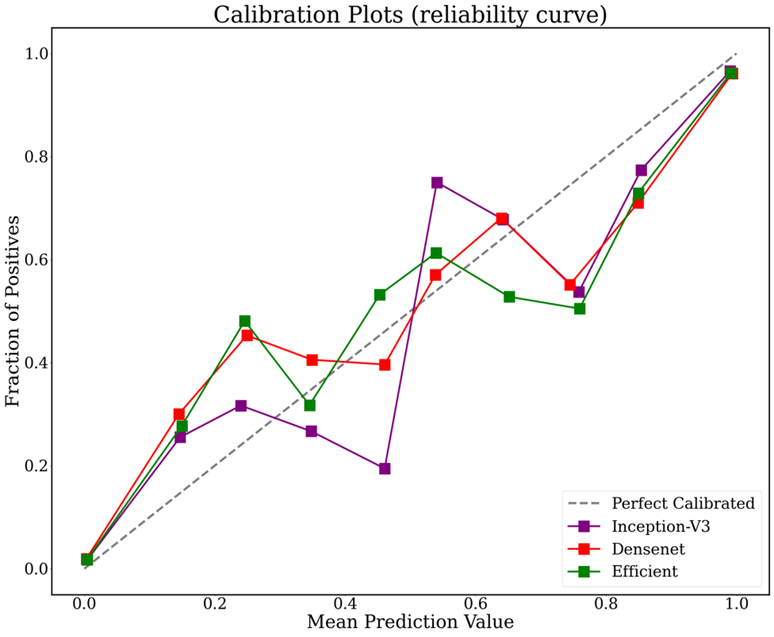


**Supplemental Figure 5**


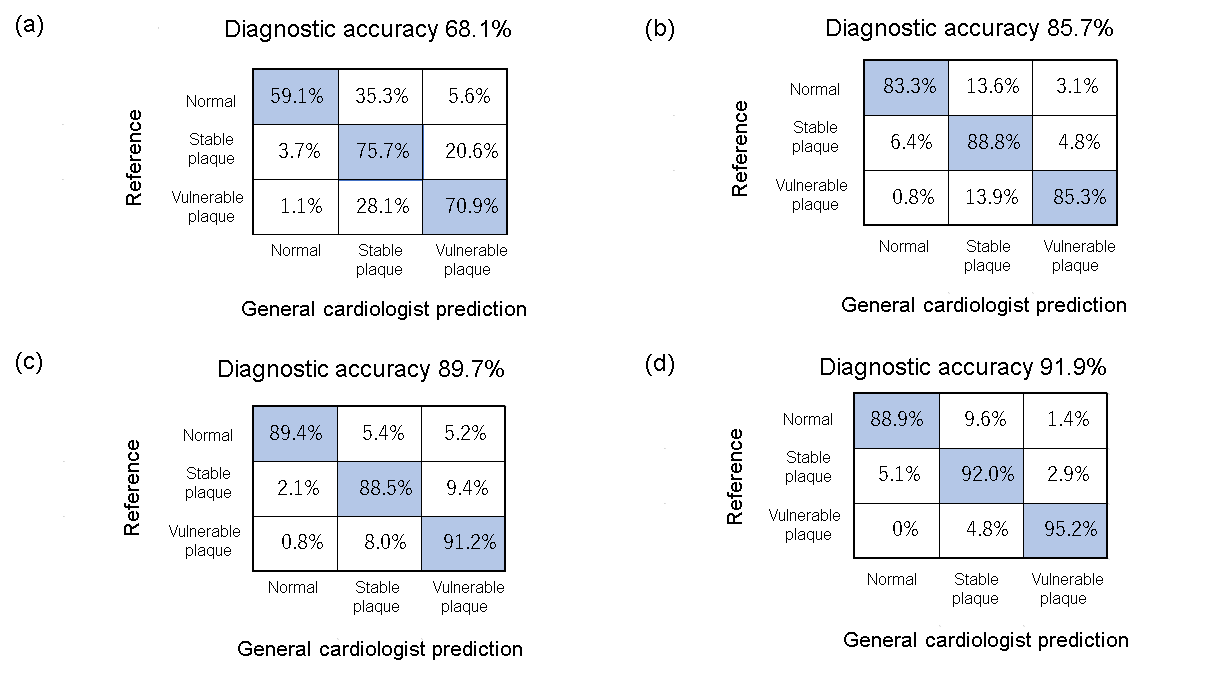


**Supplemental Figure 6**


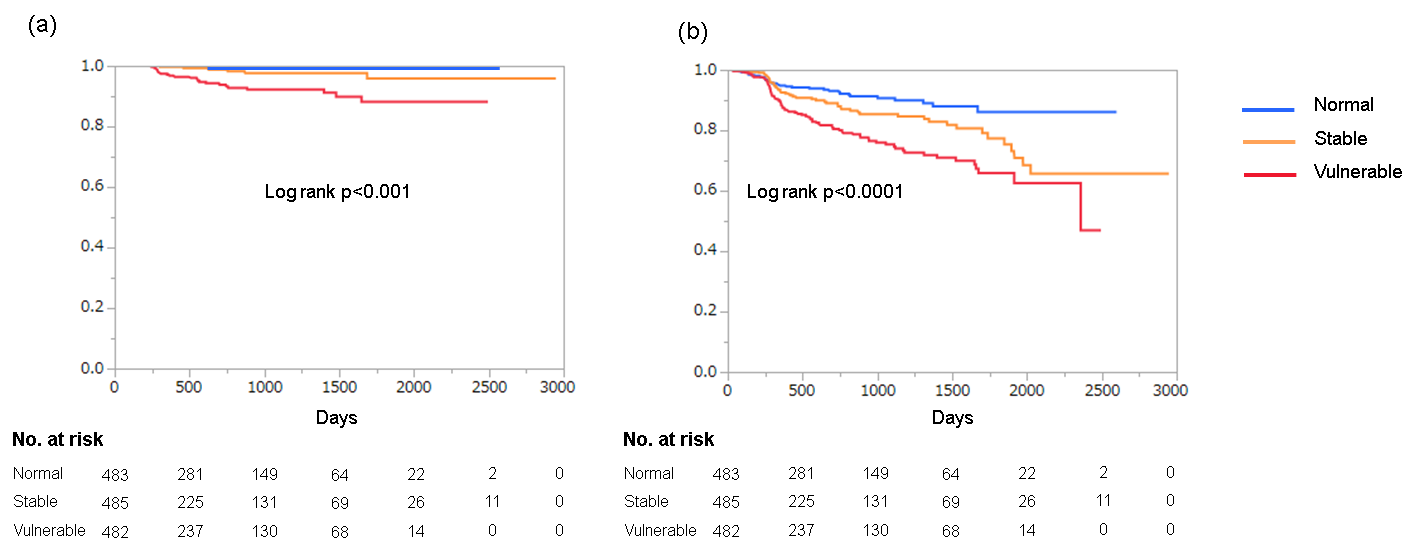

Supplement: Supplementary file 1 — Supplementary Information. [file 41598_2022_18473_MOESM1_ESM.docx]
